# *Syngonanthus androgynus*, a Striking New Species from South America, its Phylogenetic Placement and Implications for Evolution of Bisexuality in Eriocaulaceae

**DOI:** 10.1371/journal.pone.0141187

**Published:** 2015-11-11

**Authors:** Mauricio Takashi Coutinho Watanabe, Nancy Hensold, Paulo Takeo Sano

**Affiliations:** 1 Departamento de Botânica, Instituto de Biociências, Universidade de São Paulo, São Paulo, São Paulo, Brazil; 2 Keller Science Action Center, Field Museum of Natural History, Chicago, Illinois, United States of America; University of Florida, UNITED STATES

## Abstract

In the present study, we describe and illustrate a remarkable new species of *Syngonanthus* from South America (Bolivia, Brazil and Peru). This new species is quickly distinguished from all species in the genus by trimerous and bisexual flowers, a unique set of characteristics in *Syngonanthus*. Complementary to this study, sequences of 33 species were downloaded from GenBank and four species had sequences newly generated for this study. Molecular phylogenetic analyses based on nuclear ribosomal ITS and the plastid regions *psb*A-*trn*H and *trn*L-F were performed to determine its systematic position. The results have shown *S*. *androgynus* closely related to a well-supported clade that has been treated as *Syngonanthus* sect. *Carphocephalus*. Floral traits associated with this new plant also were surveyed. Character reconstruction suggests that the bisexual flowers originated independently more than once in the genus. However, trimerous flowers appear to be an ancestral condition of the whole genus.

## Introduction

Eriocaulaceae are a pantropical family consisting of 10 genera and 1200–1400 species [1,2,3,4,5]. In Brazil, various species of Eriocaulaceae have been gathered and used as dried ornamental plants for more than a century. This trade provides the main income source for hundreds of families in the Brazilian countryside [6,7]. Most Eriocaulaceae have unisexual and trimerous flowers, with bisexual or dimerous flowers uncommon [8,9]. The inflorescences are capitulum-like composed of flowers borne on a flattened receptacle at the scape apex [9], with involucral bracts at the border. The same inflorescence usually has staminate and pistillate flowers in the same capitulum [1,8,9].

*Syngonanthus* Ruhland has been recognized as the third largest genus in Eriocaulaceae [4,5]. The genus comprises approximately 120 species that exhibit a disjunct distribution in the American and African continents [1,4]. Some species of *Syngonanthus* occur in rupestrian grassland, upon shallow and sandy soils over quartzites and sandstones, like most taxa of Eriocaulaceae [3,10,11]. However, fieldwork observations have shown that some species of *Syngonanthus* also inhabit marshy or poorly drained soils [10].

Recent nomenclatural and taxonomic changes have taken place in *Syngonanthus*, including the synonymization of *Philodice* [12] and transfer of some species previously placed in *Syngonanthus* to *Comanthera*, a genus recently reestablished [13]. The current classification of the genus subdivides *Syngonanthus* into two sections: *Syngonanthus* sect. *Syngonanthus* that includes most of species and morphological diversity [14,15]; and *Syngonanthus* sect. *Carphocephalus* comprising species with elongated vegetative stems and spongy petals of staminate flowers [14].

The genus *Syngonanthus* is distinguished by the following character sets: isostemonous flowers, pistillate flowers with petals fused distally with short lobes free, petals equal or shorter than sepals, predominance of flavonoids as 6-hydroxyluteoline derivatives, and seed surface reticulate [1,4,12,14,16,17]. Other complementary characteristics are spongiose root cortex, and nectariferous and stigmatic branches diverging at the same level. Some species exhibit synflorescence axes.

In the course of preparing a revision of *Syngonanthus* sect. *Carphocephalus*, a new species came to light, that proves to be only the fourth known record of exclusively bisexual flowers in the family, and the first known occurrence of trimerous bisexual flowers. Bisexual flowers occur very rarely in the family, being known from only one trimerous andromonoecious species, *Rondonanthus flabelliformis* (Moldenke) Hensold & Giul. [18], and three dimerous, wholly bisexual species, *S*. *amazonicus* Moldenke, *S*. *trichophyllus* Moldenke, and *S*. *acephalus* Hensold. For purpose of comparison with the new species, some original updated observations on the floral morphology of these species are provided in the species discussion. The goals of the present study are to describe this new remarkable taxon and to investigate its placement in the genus. Furthermore, ancestral state reconstructions were conducted on two morphological characters related to flowers in *Syngonanthus*: merism and floral sexuality. Although more common in other genera, such as *Paepalanthus* and *Eriocaulon*, 2-parted flowers are very uncommon in *Syngonanthus*, while bisexual flowers are rare in the family as a whole.

## Material and Methods

### Ethics Statement

Vegetative tissue (leaf) for DNA analysis was donated by major collectors or extracted from herborized materials deposited in the herbaria ANDES, F, INPA and SPF. The policies for each herbarium were followed for removal of material. The samples were used only for the purpose of molecular phylogenetic study.

### Nomenclature

The electronic version of this article in Portable Document Format (PDF) in a work with an ISSN or ISBN will represent a published work according to the International Code of Nomenclature for algae, fungi, and plants, and hence the new names contained in the electronic publication of a PLOS ONE article are effectively published under that Code from the electronic edition alone, so there is no longer any need to provide printed copies.

In addition, new names contained in this work have been submitted to IPNI, from where they will be made available to the Global Names Index. The IPNI LSIDs can be resolved and the associated information viewed through any standard web browser by appending the LSID contained in this publication to the prefix http://ipni.org/. The online version of this work is archived and available from the following digital repositories: PubMed Central, LOCKSS.

### Morphological observations

The new species was described based on examination of pressed and rehydrated material under a stereomicroscope (Leica M125) and flowers were photographed for accurate observation (Leica DFC425) and measured using the software Leica Application Suite (LAS). A few flowers from each specimen with mature capitula were measured. For morphological comparison, we additionally observed under stereomicroscope other *Syngonanthus* species with similar morphological aspect, such as *S*. *weddellii* Moldenke (*S*. sect. *Carphocephalus*), and upper Amazonian species with bisexual dimerous flowers, including S. *amazonicus*, *S*. *trichophyllus* and *S*. *acephalus* (all *S*. sect. *Syngonanthus*). The DNA sample of *Syngonanthus acephalus* was identified from an image of the habit and from the sample itself. A scanning electron micrograph of the seed of *S*. *androgynus* is also presented. The study also was based on information gathered in taxonomic literature, live plants in the field and other herborized materials (F, IBGE, MO, RB, SPF).

### Sampling

For this study, both subfamilies (Eriocauloideae and Paepalanthoideae) are represented in sequences of 33 species belonging to 6 genera of Eriocaulaceae, which were downloaded from GenBank. Four additional species were newly sequenced for this work: *Syngonanthus acephalus*, *S*. *trichophyllus*, *S*. *weddellii* and the new species. The ingroup consists of a total of 31 species, representing both sections still supported for inclusion in *Syngonanthus*: *Syngonanthus* sect. *Syngonanthus* and *Syngonanthus* sect. *Carphocephalus* [4,14]. The outgroup is composed of species of *Actinocephalus*, *Paepalanthus*, *Comanthera* and *Leiothrix*. Trees were rooted with *Eriocaulon aquatile* Körn. The voucher specimen information and the GenBank accession numbers of the sequences used and generated in this study are listed in Table 1.

**Table 1:** List of the accessions used in the phylogenetic study.

Taxa	Locality	Voucher	GenBank Accession Number		
			*psb*A-*trn*H	*trn*L-F	ITS
*Actinocephalus falcifolius* (Korn.) Sano	Brazil, MG	*L*. *Echternacht 1635* (SPF)	KF840892	KF840832	KF840799
*Comanthera aciphylla* (Bong.) L.R.Parra & Giul. (1)	Brazil, MG	*M*.*J*.*G*. *Andrade* (HUEFS)	-	EU924491	EU924339
*Comanthera aciphylla* (Bong.) L.R.Parra & Giul. (2)	Brazil, MG	*L*. *Echternacht 1692* (SPF)	KF840907	-	-
*Comanthera xeranthemoides* (Bong.) L.R.Parra & Giul.	Brazil, GO	*L*. *Echternacht 1963* (SPF)	KF840906	KF840845	KF840808
*Eriocaulon aquatile* Korn.	Brazil, MG	*L*. *Echternacht 1845* (SPF)	KF840891	KF840831	KF840798
*Leiothrix angustifolia* (Korn.) Ruhland	Brazil, BA	*R*. *Harley 54660* (HEFS)	EU924364	EU924443	EU924290
*Syngonanthus acephalus* Hensold	Colombia, GUA	*M*. *Fernández 190* (ANDES)	*KT724338	-	-
*Syngonanthus androgynus* M.T.C. Watan.	Brazil, MG	*F*. *Oliveira 1056* (F)	*KT724339	*KT724342	*KT724345
*Syngonanthus anomalus* (Korn.) Ruhland	Suriname	*H*.*S*. *Irwin 55267* (P)	KF840925	KF840866	-
*Syngonanthus anthemiflorus* (Bong.) Ruhland	Brazil, MG	*L*. *Echternacht 1649* (SPF)	KF840926	KF840867	KF840818
*Syngonanthus arenarius* (Gardner) Ruhland	Brazil, MG	*M*.*J*.*G Andrade 493* (HUEFS)	EU924422	EU924498	EU924342
*Syngonanthus caulescens* (Poir.) Ruhland	Brazil, BA	*M*.*J*.*G*. *Andrade 452* (HUEFS)	EU924424	EU924500	EU924344
*Syngonanthus chrysanthus* (Bong.) Ruhland	Brazil, RS	*M*. *Trovó 115* (SPF)	KF840927	-	-
*Syngonanthus costatus* Ruhland	Brazil, MG	*L*. *Echternacht 2066* (SPF)	-	KF840871	-
*Syngonanthus cuyabensis* (Bong.) Giul., Hensold & L.R. Parra	Brazil, MT	*A*.*M*. *Giulietti 2483* (HUEFS)	EU924411	EU924489	EU924336
*Syngonanthus davidsei* Huft	Brazil, MT	*G*.*C*. *Argent 6454* (P)	-	KF840872	-
*Syngonanthus densiflorus* (Korn.) Ruhland	Brazil, TO	*M*. *Trovó 292* (SPF)	KF840930	KF840873	-
*Syngonanthus densifolius* Silveira	Brazil, MG	*L*. *Echternacht 1689* (SPF)	KF840931	KF840874	-
*Syngonanthus fischerianus* (Bong.) Ruhland	Brazil, SP	*M*. *Trovó 171* (SPF)	KF840934	KF840877	KF840820
*Syngonanthus flavidulus* (Michx.) Ruhland	United States, NC	*J*.*R*. *Masssey 3284* (P)	KF840932	KF840875	-
*Syngonanthus gracilis* (Bong.) Ruhland	Brazil, MG	*L*. *Echternacht 1709* (SPF)	KF840936	KF840879	KF840822
*Syngonanthus helminthorrhizus* (Mart. ex Korn.) Ruhland	Brazil, SP	*M*. *Trovó 315* (SPF)	KF840937	KF840880	KF840823
*Syngonanthus heteropeplus* (Korn.) Ruhland	Brazil, AM	*F*.*A*. *Carvalho 1443* (INPA)	KF840945	KF840888	KF840828
*Syngonanthus laricifolius* (Gardner) Ruhland	Brazil, MG	*L*. *Echternacht 1870* (SPF)	KF840938	KF840881	KF840824
*Syngonanthus longipes* Gleason	Brazil, AM	*F*.*A*. *Carvalho 1831* (INPA)	KF840939	KF840882	-
*Syngonanthus macrolepis* Silveira	Brazil, MG	*L*. *Echternacht 1902* (SPF)	KF840940	KF840883	KF840825
*Syngonanthus minutulus* (Steud.) Moldenke	Brazil, MG	*L*. *Echternacht 1797* (SPF)	KF840935	KF840878	KF840821
*Syngonanthus niger* Silveira	Brazil, MG	*L*. *Echternacht 1840* (SPF)	KF840941	KF840884	-
*Syngonanthus nitens* (Bong.) Ruhland	Brazil, MG	*L*. *Echternacht 1815* (SPF)	KF840942	KF840885	KF840826
*Syngonanthus peruvianus* Ruhland ex Ule	Peru	*P*.*C*. *Hutchison 556* (P)	KF840928	KF840870	KF840819
*Syngonanthus poggeanus* Ruhland	DR Congo	*Duvigneaud 1342* (BRLU)	KF840943	KF840886	-
*Syngonanthus reclinatus* (Korn.) Ruhland	Brazil, TO	*L*. *Echternacht 2118* (SPF)	KF840944	KF840887	KF840827
*Syngonanthus trichophyllus* Moldenke	Brazil, RR	*F*.*A*. *Carvalho 1580B-B* (INPA)	*KT724340	*KT724343	*KT724346
*Syngonanthus umbellatus* (Lam.) Ruhland	Brazil, AM	*L*. *Echternacht 2097* (SPF)	KF840946	KF840889	KF840829
*Syngonanthus weddellii* Moldenke	Brazil, TO	*M*. *Watanabe 381* (SPF)	*KT724341	*KT724344	*KT724347
*Syngonanthus widgrenianus* (Korn.) Ruhland	Brazil, MG	*L*. *Echternacht 1952* (SPF)	KF840947	KF840890	KF840830
*Syngonanthus williamsii* (Moldenke) Hensold	Brazil, AM	*F*.*A*. *Carvalho 2208b* (INPA)	KF840948	-	-
*Paepalanthus bryoides* (Bong.) Kunth (1)	Brazil, MG	*F*.*N*. *Costa 263* (HUEFS)	EU924373	-	-
*Paepalanthus bryoides* (Bong.) Kunth (2)	Brazil, MG	*L*. *Echternacht 1804* (SPF)	-	KF840834	KF840801

List of investigated species used in this study with species Author, locality (country and major political subdivision, when available and applicable), voucher information (collector number and herbarium) and GenBank acession numbers for three regions (*psba*A-*trn*H, *trn*L-F and ITS).

^-^The region was not sequenced for the taxon.

*Newly sequences generated for this study.

### Molecular markers

For phylogenetic reconstruction in *Syngonanthus* and investigation of the placement of the new taxon, we analyzed data from DNA sequences of the internal transcribed spacer region of the nrDNA tandem repeat (ITS) and plastid *psb*A-*trn*H and *trn*L-F regions. Genomic DNA from silica-gel dried tissue or herbarium specimens were extracted using a modified 2X cetyl trimethylammonium bromide (CTAB) protocol [19]. To generate sequences used in this study we amplified the DNA regions using polymerase chain reaction (PCR) protocols previously used for Eriocaulaceae [16,20]. The chromatograms were assembled and edited with Geneious software (Geneious Pro v7.1.7 software Biomatters Ltd., Auckland, New Zealand).

### Sequence alignment, phylogenetic analysis and character reconstruction

Alignment of the DNA sequences was initially carried out using MAFFT v.7 [21] followed by minor manual adjustments in Geneious. The alignment is available at Figshare repository (http://dx.doi.org/10.6084/m9.figshare.1526094). Echternacht et al. [16] evaluated congruence among trees resulting from analyses of the same molecular markers used in our study and no conflicts were identified. We analyzed combined data sets conducting Bayesian inference (BI) analyses as implemented in MrBayes 3.2.3 [22]. The best-fit model of nucleotide evolution for Bayesian analyses was chosen separately for each marker using JModelTest 2 [23]. The models were selected using Akaike information criteria (AIC) for all data sets. The best models fitted were TPM1uf + I + G for *psb*A-*trn*H, TVM + I + G for *trn*L-F, and GTR + I + G for ITS. The combined analysis consisted a partitioned data with each marker decoupled. The heuristic search was composed by two independent runs of four Markov chains Monte Carlo (MCMC) running 10 million generations, sampling results every 1,000 generations. Trees were discarded as burn-in (25%) of the sampled trees after checking the stability using Tracer v1.6 [24]. Branch support was assessed by Bayesian posterior probabilities (PP). The majority rule consensus tree and PP were determined by combination of two runs using the trees sampled after the convergence of the chains.

The evolution of floral characters in *Syngonanthus* was inferred by estimating the marginal posterior probability of ancestral states using a Bayesian framework [25]. This approach allows us to incorporate phylogenetic uncertainty during the inference. Ancestral states were estimated using BayesTraits V2 [26] with a set of 1000 post-burn-in samples of trees. Transition rates were set to be equal and a hyperprior with exponential distribution was used to estimate the rate coefficient. Ancestral states for nodes with posterior probabilities < 1.0 were estimated using the MRCA command [25,26]. The MCMC was run for 10,000,000 generations, with the first 2,500,000 generations discarded as burn-in (25%). Values were saved for every 1000^th^ generation. A total of 7,500 generations were saved and used to compute mean values of frequency for each character state. Two characters were examined: floral merism: (0) dimerous, (1) trimerous; and floral sexuality: (0) unisexual, (1) bisexual.

## Results and Discussion

### Taxonomic treatment

**Fig 1:**
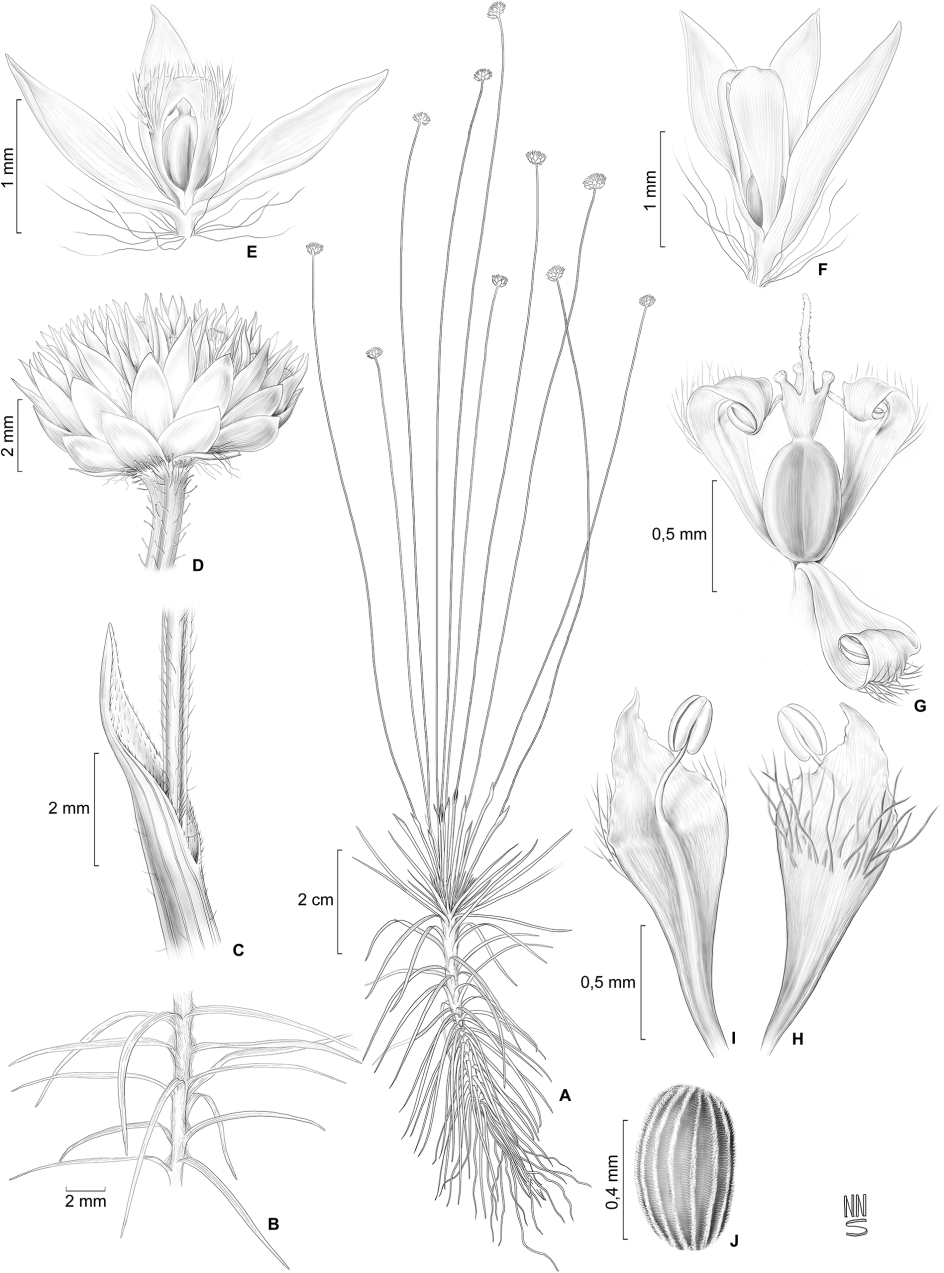
Illustration of *Syngonanthus androgynus*. (A) Habit. (B) Distribution of leaves on aerial stem. (C) Details of spathe and scape. (D) Capitulum. (E-F) Flower variation: (E) Flower with shorter and pilose corolla. (F) Flower with longer and glabrous corolla. (G) Dissected flower showing gynoecium and involute petals enclosing anthers and two style branches. (H) Petal abaxial view. (I) Petal adaxial view. (J) Seed. Drawn by Natanael Nascimento from *Fonseca & Filgueiras 115* (A, C-E, G-J) and *Albán Castillo & Foster 6968* (B, F).

**Fig 2:**
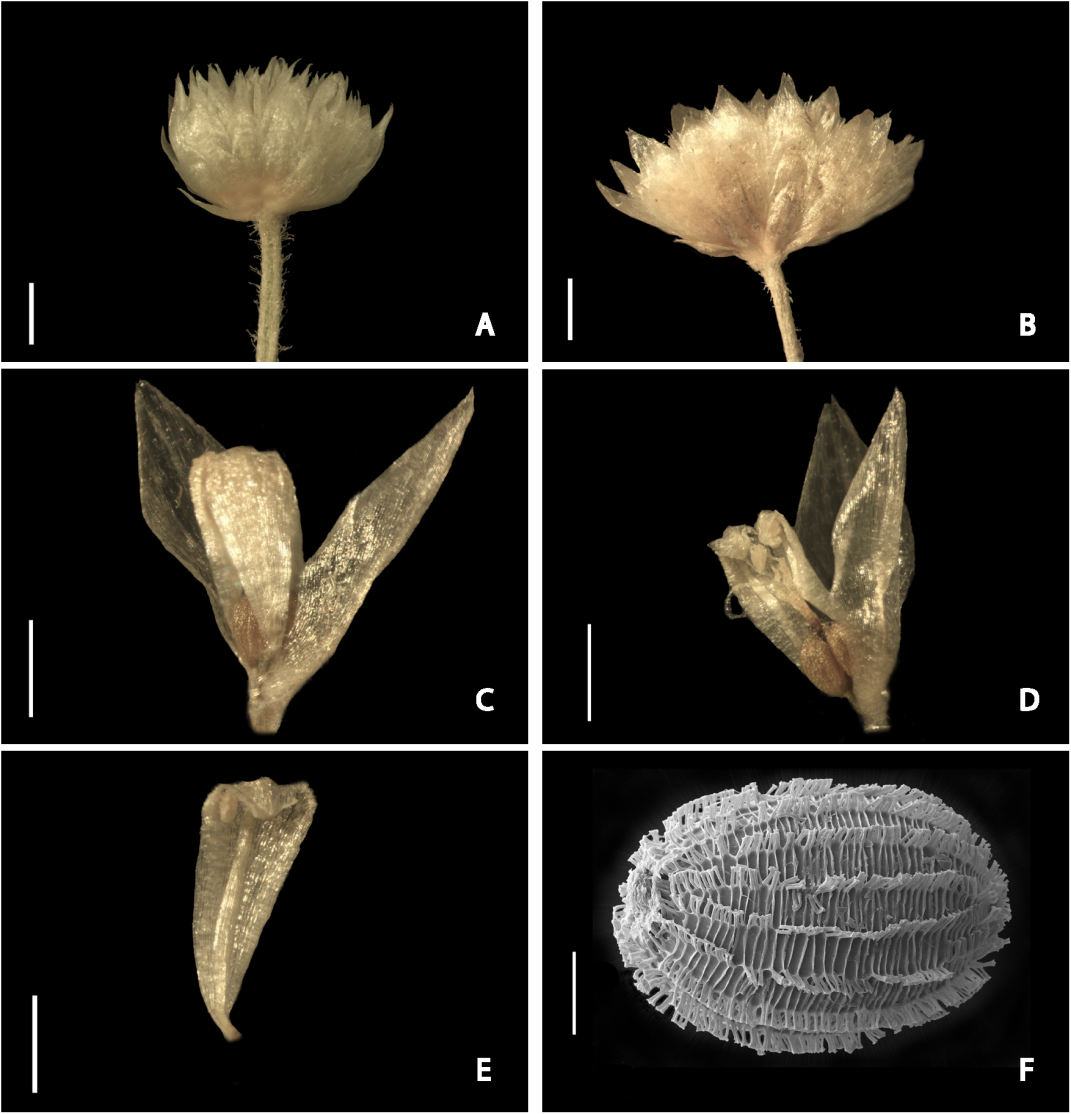
*Syngonanthus androgynus* details. (A-B) Capitula. (A) Specimen from Brazil. (B) Specimen from Peru. (C) Bisexual flower with one sepal removed. (D) Dissected bisexual flower with one sepal and one petal removed. (E) Petal with evidently adnate filament. (F) Seed. Scale bars: 1 mm (A-B); 0.5 mm (C-E); 100 μm (F), Photos from *Oliveira et al*. *1056* (A), *Albán Castillo & Foster 6968* (B-E) and *Diáz et al*. *9201* (F).

***Syngonanthus androgynus*** M.T.C.Watan. ***sp*. *nov*.** [urn:lsid:ipni.org:names: 77150340–1] (Figs 1 and 2). Type: BRAZIL, Goiás: Alto Paraíso de Goiás, Chapada dos Veadeiros, Km 11 da estrada Alto Paraíso de Goiás / São Jorge, estrada para a cachoeira São Bento, ca. 1.5 km após a cachoeira, 09 Sep 1994, M. Fonseca & T. Filgueiras 115 (holotype, SPF; isotypes, IBGE, RB).

*Syngonanthus androgynus differs from all described Syngonanthus by the exclusively trimerous and bisexual flowers*. *This new species has a similar habit to Syngonanthus weddellii but differs by lax and decurved leaves on aerial stem (versus congested and patent leaves)*.

Caulescent herbs, 12–37 cm tall, unbranched at the base, lacking basal rosette. Roots white, 0.1–0.2(–0.6) mm in diameter, spongy. Aerial stem 2–13 cm long, villous with white, crooked trichomes mainly concentrated near the apex, bearing an umbel of (1–)3–30 inflorescences at the apex. Leaves abundant, spirally arranged and equally distributed on the stem, ascending, lax, sometimes more congested at the apex near the synflorescence, decurved, 0.5–4.5 cm × 0.25–2.00 mm, linear to threadlike, capillary, or filiform, acuminate, both surfaces pilose with capitate filamentous hairs and adpressed trichomes, later glabrescent, veins 3–6 prominent. Leaves at stem apex more congested (bracts), 0.3–2.0 cm long, linear or acicular, the apex recurved and rigid, pubescent with adpressed trichomes and capitate hairs. Spathes 1.0–5.5 cm long, chartaceous, cylindrical, erect, obliquely opened, tip incurved, triangular, acute or cuspidate at apex, margin entire, ciliate, pilose with capitate and filamentous trichomes abaxially and at adaxial apex, otherwise glabrous. Scapes erect, 3–34 cm long, greenish or straw-colored to golden with age, 3–costate, pilose throughout with filamentous hairs, pubescent near the apex with capitate hairs and adpressed trichomes. Capitula 3–7 mm in diameter, radiate, hemispherical at anthesis. Involucral bracts in 2–5 series, cream-colored, all similar in color or sometimes the outer castaneous, the inner cream, hyaline when wet, glabrous, those of the external series 1.05–1.55 × 0.50–0.90 mm, varying from triangular to slightly ovate, obovate or oblong, apex acute or obtuse, the inner bracts are progressively narrower, those of the internal series 1.0–1.8 × 0.50–0.75 mm, almost equaling the flower height, oblanceolate, apex obtuse to rounded, sometimes notched; receptacle discoid, pilose. Floral bracts absent. Flowers bisexual, 3-merous, 20–60 per capitulum, 1.45–2.55 mm long including pedicels; pedicels 0.15–0.45 mm long, with long soft, crooked, white trichomes at base and insertion with receptacle; sepals 1.5–2.0 mm long, membranous, chartaceous toward apex, glabrous, white to cream, hyaline when hydrated, slightly white in a medial longitudinal band, shortly connate at base, lanceolate, rhombic or narrowly obtrullate, apex acute, margin involute; petals 1.0–2.2 mm long, rhombic, connate at the upper margin, free at base and apex, lobes triangular, apex acute, lower half of petal fleshy, upper half membranous, pilose abaxially with simple filamentous hairs, involute after anthesis; filaments adnate to the petals at base, anthers 0.15–0.30 mm long, white; gynoecium 1.3–1.5 mm long; ovary 0.50–0.75 mm long; style column ca. 0.1–0.2 mm long, cream-colored; appendages ca. 0.15–0.30 mm long, the glandular apex capitate; style branches 0.5–1.0 mm long, broad and membranous at base, apex narrow and papillose. Fruit a dehiscent capsule; seeds 0.45–0.60 × 0.25–0.35 mm, ovoid, brown-colored, striate with short pseudotrichomes in longitudinal lines.

### Etymology

The specific epithet refers to the bisexual flowers, in which androecia and gynoecia are both fully developed, a very uncommon characteristic in the family.

### Habitat, distribution and phenology

*Syngonanthus androgynus* has a disjunct distribution. It has been collected in the Amazonian region along the border of Bolivia and Peru in the floodplain of the Río Heath. Two other populations were detected over 2000 km away, in the Grande Sertão Veredas National Park and near Chapada dos Veadeiros National Park (Central Brazil). This species grows in wetlands, over shallow sandy soils in Bolivian Amazon “Pampas” vegetation and in the Brazilian cerrado. It was observed in the field in terrestrial habitats, but may be facultatively aquatic, since it is also recorded from seasonally inundated habitats (*M*. *Watanabe et al*. *409*) and permanent flooded areas (*C*. *Diáz et al*. *9201*). Flowers have been recorded from May-September, abundant fruit from September (*C*. *Diáz et al*. *9201*).

### Conservation status

Although *S*. *androgynus* also occurs in a protected area at Grande Sertão Veredas National Park, few populations known within very limited range allow categorization of this species as endangered following the criterion B (geographic range) of World Conservation Union Red List Categories and Criteria [27]. Area of occupancy is < 500 km^2^ (calculated using GeoCAT [28]), and number of locations ≤ 5 accord with the requirements of criterion B2ac.

### Species recognition

*Syngonanthus androgynus* is the first known species of Eriocaulaceae to have exclusively bisexual, trimerous flowers. *Rondonanthus flabelliformis* was originally described as such but was later found to be andromonoecious, with staminate and bisexual flowers mixed in the same capitulum [18]. Only three other species, *S*. *acephalus*, *S*. *amazonicus*, and *S*. *trichophyllus*, from the Guianas and adjacent parts of the upper Amazon basin, have been found to have exclusively bisexual flowers, but these are dimerous. *Syngonanthus acephalus* is a very unusual moss-like cushion-plant with capitula reduced to one or two flowers borne at the stem apex and lacking involucral bracts. The other two species have unusual conical capitula, with large scale-like receptacular bracts subtending the flowers. The flowers of *S*. *trichophyllus*, it may be noted, were tentatively described, from senescent, half-shattered capitula, as 3-merous and unisexual, with staminate flowers not seen [29]. However, examination of the type and numerous other specimens shows that the flowers are consistently 2-merous and similar to those of *S*. *amazonicus* except for the reduction of anthers from two to one in *S*. *trichophyllus*. In addition, in both species the flowers are bilaterally symmetric with the two sepals spathaceously fused in the adaxial position, opposite the floral bract (Hensold, unpublished). (Selected vouchers examined of *S*. *trichophyllus*: Colombia, *Garcia-Barriga & Schultes 14138* (LL), holotype; Brazil, *Prance 29118* (MO); Colombia, *P*. *Franco 3653* (F); Guyana, *K*.*Wurdack et al*. *5078* (F); Venezuela, *B*. *Stein 1511* (F)).

*Syngonanthus androgynus* in comparison has 3-merous radially symmetric flowers with sepals only briefly connate, and floral bracts lacking. Morphologically they are similar to the pistillate flowers of *S*. *weddellii* Moldenke. Both species have the same texture and form of petals: connate at the upper margin, free at base and very top, lower half of petal fleshy, upper half membranous. *Syngonanthus androgynus* is distinguished from *S*. *weddellii* by the characteristics mentioned in the *diagnosis*. *S*. *weddellii* was only known from the type collection but was rediscovered recently by the first author in Tocantins state (Brazil).

This new species could be the first reported case of cleistogamy in the Eriocaulaceae. The petals are hooked inward over each other at the apex in all stages observed, apparently never completely opening, and the stamens and style branches are wrapped around each other inside the flower. Petals are not completely fused at the top but strongly inflexed-uncinate completely enclosing the sexual parts. The collection from Chapada dos Veadeiros chosen as the holotype includes well-developed individuals, while specimens from Grande Sertão Veredas National Park (ca. 200 km distant) comprise smaller and juvenile plants. Collections from the Río Heath usually have leaves scattered on aerial stem but congested apically near scapes, unlike those from Central Brazil that have leaves longer, more congested and more equally distributed over the length of the stem. Plants from Río Heath also differ by the petals of pistillate flowers glabrous, while those from Brazil have petals of pistillate flowers pilose in most cases, rarely glabrescent. The flowers are also slightly flattened and stamens larger in the Brazilian plants. We do not treat these as two different taxa because of the small sample size and the inherent variability of wetland species. In addition, the floral structure and vegetative arrangement is very similar in all specimens and the pilosity in the flowers as well as the distribution of leaves on stem also showed slight gradation within plants from Brazil.

Additional specimens examined (Paratypes). BOLIVIA. La Paz, Abel Iturralde, Puerto Muscoso, donde llega la pampa mas cerca del río Heath, 25 July 1995, *N*. *Helme & L*. *Kruger 678* (F). BRAZIL. Minas Gerais, Formoso, Parque Nacional Grande Sertão Veredas, próximo a ponte da barra do Rio Preto / Santa Rita, em vereda, 15°10’35”S 45°46’09”W, 21 May 1998, *F*. *Oliveira et al*. *1056* (F, IBGE); Chapada Gaúcha, Parque Nacional Grande Sertão Veredas, em borda de lagoa, próximo ao córrego da onça, 19 July 2015, *M*. *Watanabe et al*. *409* (SPF). PERU. Madre de Dios, Tambopata, Parque Nacional “Bahuaja-Sonere”, ex Santuario Nacional “Pampa de Heath”, formación de pampa inundada permanentemente, 25 September 1997, *C*. *Diáz et al*. *9201* (F, MO); Río Heath, Santuario Nacional de las Pampas del Heath, lado este de la Pampa, 3–4 km oeste del río, camino del Refugio Juliaca, hasta el bosque de galería y campamento Aguas Claras, 15 June 1992, *J*. *Albán Castillo & R*. *Foster 6968* (F).

### Phylogenetic analysis

**Fig 3:**
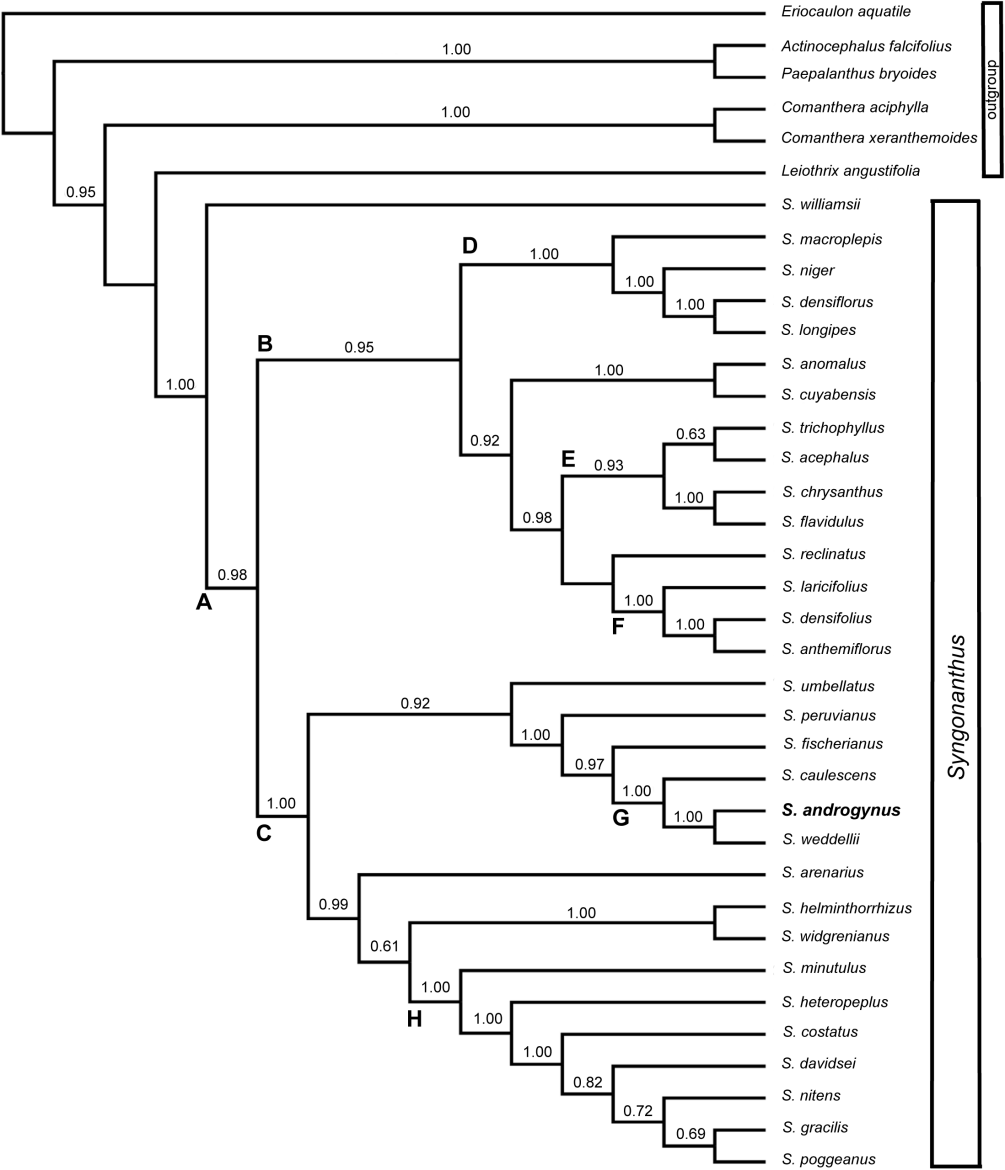
Phylogenetic tree. Cladogram of Bayesian analysis of the combined plastid and nuclear data (*psb*A-*trn*H, *trn*L-F and ITS) showing the position of *Syngonanthus androgynus*. Posterior probabilities more than 0.60 are shown above the branches.

Sequences for ITS (nuclear), *trn*L-F and *psb*A (both plastid loci) were combined for 37 accessions (31 ingroup and 6 outgroup). The final alignment comprised 2440 pb. Some named (A-H) nodes in Fig 3 received high statistical support (except clade E, moderate).

Our molecular phylogenetic results show *Syngonanthus* strongly supported (PP 1.00) and monophyletic, corroborating previous analysis in the family [4,16]. *Syngonanthus williamsii* appears as the early divergent lineage within *Syngonanthus* and sister of clade A, which contains the remaining species of the genus (PP 0.98). *Syngonanthus williamsii* is a distinctive species of the genus with morphological characteristics rare in *Syngonanthus* such as the developed synflorescence axis covered along its length with foliaceous bracts and leaves of the rosette with apex bifid.

Clade A is composed of two main clades B and C with high support (PP 0.95 and 1.00, respectively). Here it is demonstrated that bisexual flowers emerged independently from two distinct lineages within *Syngonanthus*. Although the clade containing *S*. *acephalus* and *S*. *trichophyllus* has weak support (PP 0.63), it becomes evident that the ancestor of this group came from the lineage of clade B, whereas the ancestor of *S*. *androgynus* came from clade C.

Clade D (PP 1.00) is a well-supported clade, comprising robust species with thick synflorescence axes, including *S*. *macrolepis* as basal species. This clade also shows *S*. *niger* as sister to a well-supported clade with *S*. *longipes* and *S*. *densiflorus* (PP 1.00).

Clade E has moderate to high statistical support (PP 0.93) consisting of two subgroups: the “bisexual dimerous species” clade, consisting of *S*. *trichophyllus* and *S*. *acephalus* as sister species (PP 0.63); and a subgroup consisting of *S*. *chrysanthus* and *S*. *flavidulus* (PP 1.00). Clade F (PP 1.00) is strongly supported and consists of a group of *Syngonanthus* from *Cadeia do Espinhaço* (mainly in Minas Gerais State, Brazil). This small group shares a conspicuous character of showy and dimorphic involucral bracts.

Clade G includes species treated under *Syngonanthus* sect. *Carphocephalus* (excluding *S*. *anomalus*) and it is well supported (PP 1.00). Here, *S*. *caulescens* is the first divergent species and *S*. *androgynus* appears as sister of *S*. *weddellii*. This taxonomic placement of *S*. *androgynus* (within *Syngonanthus* sect. *Carphocephalus*) corroborated our original hypothesis. The new species shares characteristics observed in this group like fleshy petals, and well-developed aerial stem with leaves spirally distributed.

The phylogenetic analysis also reveals multiple origins for bisexual flowers in *Syngonanthus*. *Syngonanthus trichophyllus* and *S*. *acephalus* probably share the same ancestor for bisexual flowers and they are most related to plants from clade E and F. It is surprising because clades E (especially regarding *S*. *trichophyllus* and *S*. *acephalus*) and F are groups with distinct morphological structure, inflorescence and geographical distribution.

Clade H shows a known strong group (PP 1.00) that consists of rosette plants without synflorescence axes, such as *S*. *minutulus*, *S*. *heteropeplus*, *S*. *costatus*, *S*. *davidsei*, *S*. *nitens*, *S*. *gracilis* and *S*. *poggeanus*.

### Character reconstruction

**Fig 4:**
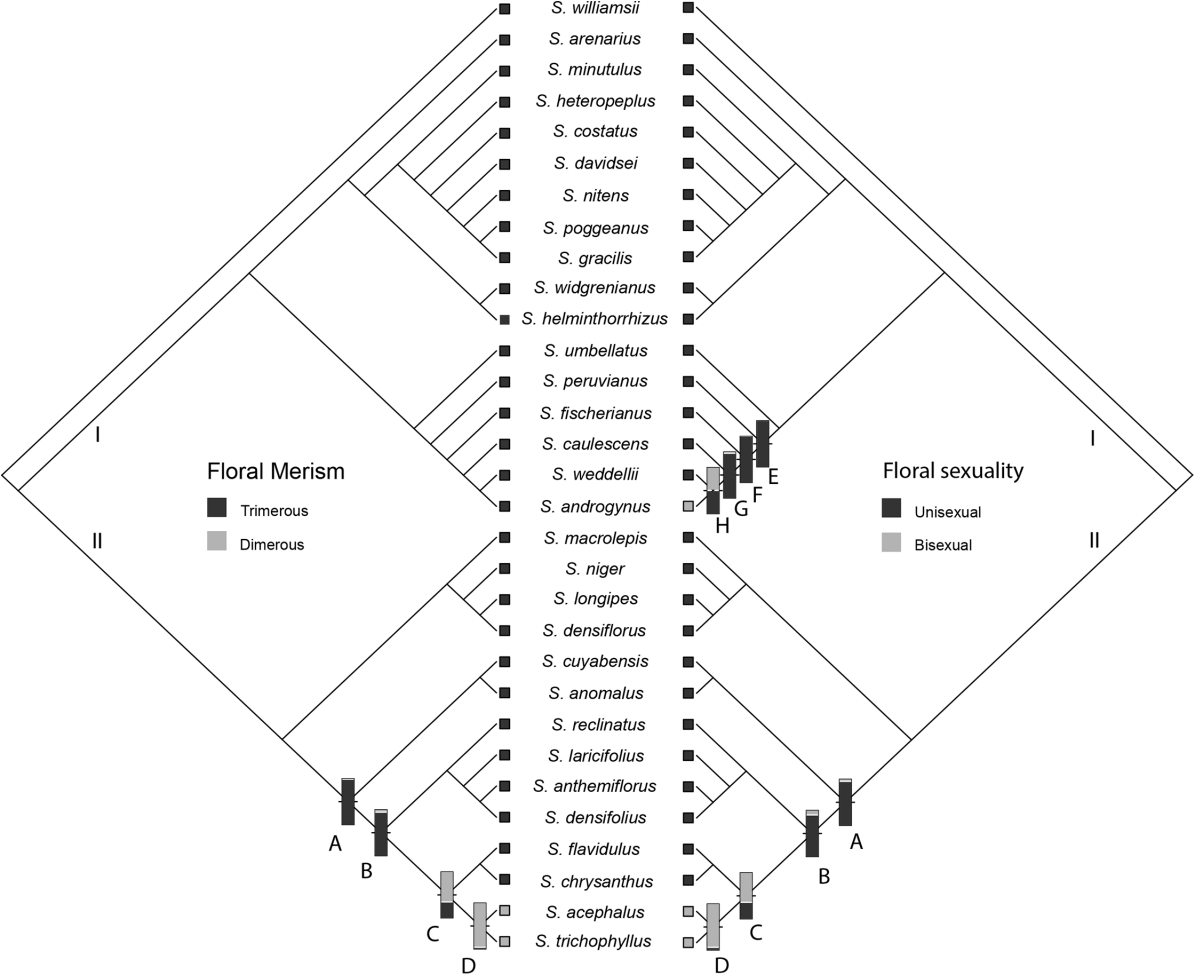
Ancestral character reconstruction. Ancestral character reconstruction for floral traits (merism and floral sexuality) based on the Bayesian tree inference of the combined plastid and nuclear data (*psb*A-*trn*H, *trn*L-F and ITS), considering only *Syngonanthus* species.

Probabilities of the ancestral state reconstruction of the nodes with most interest for our analysis in *Syngonanthus* are provided in the Fig 4. Informally, we subdivided the cladogram (I and II), for easy viewing of the two lineages of the genus that emerged in our results, and to show that the shifts occurred independently in the less inclusive clades.

Monocots usually are characterized by trimerous-pentacyclic flowers [30]. Most species of Eriocaulaceae have trimerous flowers, but dimerous species are found in *Eriocaulon*, *Lachnocaulon*, *Paepalanthus*, *Comanthera* and *Syngonanthus* [1,8,9,31,32,33]. All of these genera are monophyletic groups, except *Paepalanthus* [2,4,16,20] implying that dimerous flowers exhibit a considerable homoplasy in the family with multiple origins. Our reconstruction resulted in trimerous flowers as ancestral character state for *Syngonanthus* (Fig 4). Dimerous flowers, a character state shared by *S*. *acephalus* and *S*. *trichophyllus*, appears to be a synapomorphy of this clade (D). *Syngonanthus amazonicus*, another species with dimerous flowers in the genus, was not sampled in our molecular analysis, but morphological evidence and similarity with *S*. *trichophyllus* suggest this taxon probably belongs to this clade (See Species Recognition). There is only one other species with 2-merous flowers in the genus, *S*. *minutus* (Moldenke) Hensold, a species known only from the type [31]. Probably, *S*. *minutus* is not related with *S*. *acephalus* / *S*. *trichophyllus*, although it also has dimerous flowers. *Syngonanthus minutus* has unisexual flowers, with petals free in both sexes, rosulate habit and falcate asymmetric calyces of the staminate flowers, a set of features suggesting close relationship with a group of *Syngonanthus* which includes *S*. *gracilis* (Bong.) Ruhland [31] (Fig 3, clade H). Although we have not sampled *S*. *minutus* in the molecular phylogeny, it is speculated that 2-parted flowers also are a homoplastic character within *Syngonanthus*.

Eriocaulaceae have unisexual flowers, with rare exceptions of bisexual flowers in the family, which could be considered a reversal state in Poales [34]. The bisexual condition of the flowers appears to have originated independently twice in the genus, at least (Fig 4, clade D and H). This scenario makes sense, since the bisexual-flowered species form two morphologically very distinctive groups, with distinctive origins (Clade I and II): *S*. *androgynus* has 3-parted ebracteate regular flowers, and *S*. *acephalus* and *S*. *trichophyllus* are dimerous and bilaterally symmetrical, enclosed by large bracts. The ancestral state reconstruction for floral sexuality revealed that unisexual flowers represent the ancestral character state in *Syngonanthus*, which is not surprising since most Eriocaulaceae have unisexual flowers. The bisexual condition of *S*. *androgynus* seems to be an isolated transition in *Syngonanthus* sect. *Carphocephalus*, while *S*. *acephalus* and *S*. *trichophyllus* may share a common ancestor with this floral trait. In summary, the evolution of bisexual flowers in Eriocaulaceae appears to have had multiple origins, including as well the emergence of this feature in *Rondonanthus*, specifically in *R*. *flabelliformis*, another homoplastic occurrence of this character.

## Supporting Information

S1 Fig Posterior probability distributions of the reconstructed ancestral states. Posterior probability distributions of the reconstructed ancestral states at selected nodes as named on the trees in the Fig 4. Values given are the means for the distribution of each character state.
(PDF)
