# Supplementary figures and images for: Syngonanthus androgynus, a Striking New Species from South America, its Phylogenetic Placement and Implications for Evolution of Bisexuality in Eriocaulaceae
Source: PLoS One. 2015 Nov 11;10(11):e0141187. doi: 10.1371/journal.pone.0141187 (PMC4641623; doi:10.1371/journal.pone.0141187)

# Floral Merism

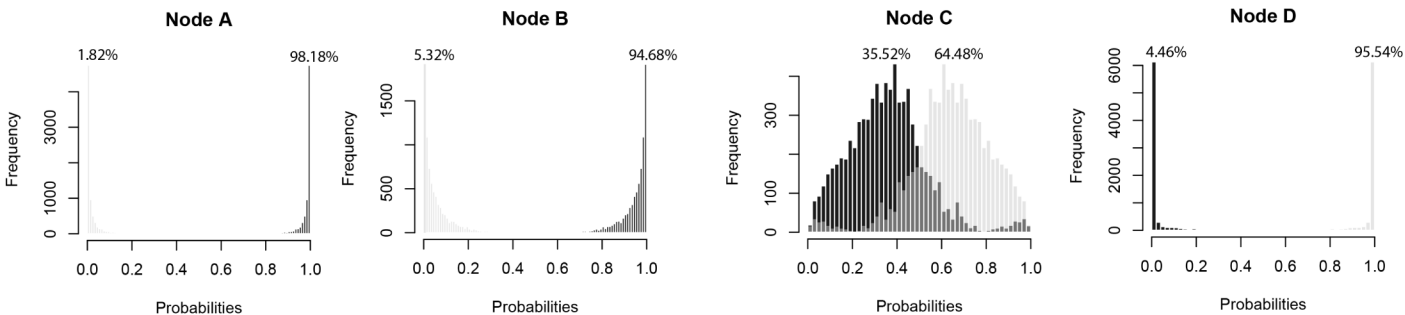

# Floral bisexuality

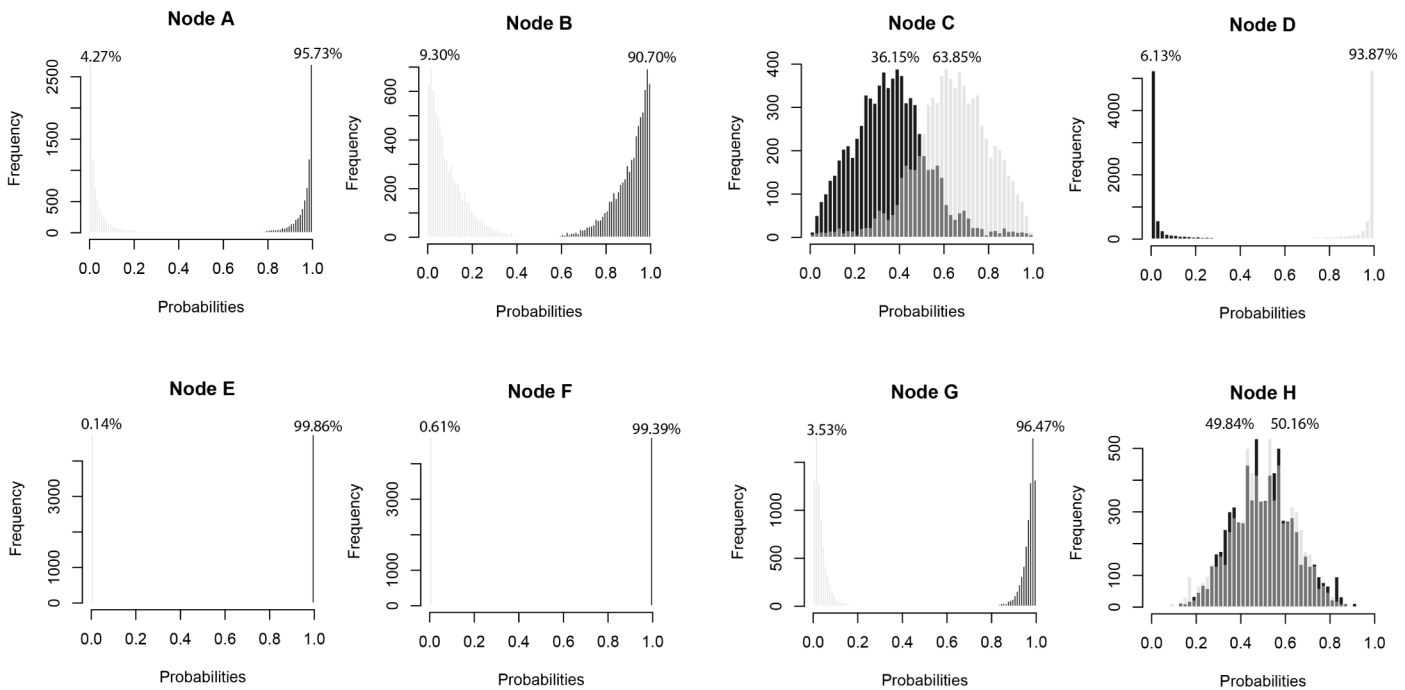

Supplement: S1 Fig — Posterior probability distributions of the reconstructed ancestral states at selected nodes as named on the trees in the Fig 4. Values given are the means for the distribution of each character state. (PDF) [file pone.0141187.s001.pdf]
